# Using the iCasp9 suicide strategy to control the growth and function of genome-edited B cells with redirected antigen specificity

**DOI:** 10.1016/j.omton.2025.201104

**Published:** 2025-11-22

**Authors:** Jenny Léonard, Marine Cahen, Anne-Laure Tanguy, Laurent Deleurme, Natsuko Ueda, Ophélie Dézé, Grégory Noël, Maiwenn Pineau, Christophe Ferrand, Yannic Danger, Michel Cogné

**Affiliations:** 1 INSERM UMR1236, University of Rennes, Etablissement Français du sang, 35000 Rennes, France; 2 INSERM UMR1098, EFS BFC, Université de Bourgogne Franche-Comté, 25000 Besançon, France

**Keywords:** MT: Regular Issue, B cell, adoptive immunotherapy, suicide gene, genome editing, engineered B cells

## Abstract

B cells could be effective immunotherapeutic “drug cells,” but reports of genomic editing to redirect their specificity have not included safety strategies. To address the potential complications of cell therapy, there is a growing demand for integrated safety switches. This is particularly pertinent in the case of B cells, which are prone to malignant transformation. We evaluated in B cells the efficacy of inserting the inducible caspase-9 (iCasp9) suicide gene, together with either a reporter gene or a single-chain immunoglobulin cassette specific for a tumor antigen. We demonstrate that a single edit of the IgH locus enables the expression of both iCasp9 and the cassette hijacking antigen specificity, while preserving B cell functionality. In both primary and malignant lymphoma B cells, activation of iCasp9 using the drug AP1903 readily induced apoptosis of edited cells, both *in vitro* and in established tumors grafted to immunodeficient animals. Although AP1903 treatment strongly curbed edited cell survival, this was constantly followed by the selection of resistant cells with lowered expression of both iCasp9 and the therapeutic antibody cassette. Therefore, in adoptive immunotherapy protocols, the iCasp9/AP1903 safety switch could stand as an efficient neoadjuvant therapy, as well as a rheostat to modulate the infusion of a therapeutic molecule.

## Graphical abstract

**Figure:**
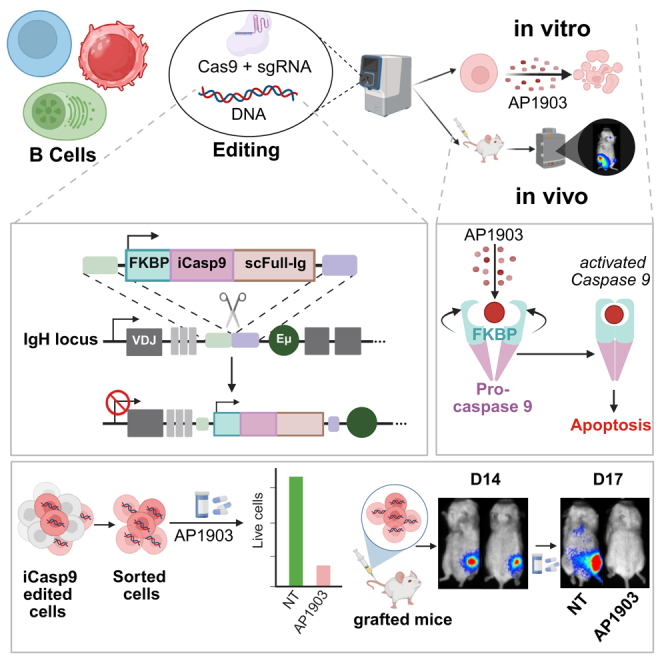

## Teaser

B cells can serve as immunotherapeutic drug cells, but require integrated safety switches due to oncogenic risks. Inserting an inducible caspase-9 (iCasp9) suicide gene enables AP1903 drug-induced apoptosis of edited B cells *in vitro* and *in vivo*, providing a means to control therapy and potentially modulate antibody infusion via the iCasp9/AP1903 system.

## Introduction

The development of CRISPR-Cas9 technology has revolutionized the field of genome engineering, paving the way for the transformation of immune cells into “drug cells” through targeted editing of their specific receptor genes.^1^ Adoptive immunotherapy, which harnesses the therapeutic potential of such modified cells, has already achieved major therapeutic breakthroughs with chimeric antigen receptor (CAR-T)-modified T cells, whose clinical applications are expanding.^1^^,^^2^^,^^3^ In parallel, similar procedures are beginning to emerge for NK cells, dendritic cells, and, more recently, B cells.^4^^,^^5^^,^^6^^,^^7^^,^^8^^,^^9^^,^^10^^,^^11^

B lineage cells are optimal Ig producers. Adoptive immunotherapy strategies using edited B cells would offer the potential for durable immune memory, sustained therapeutic antibody delivery, targeting tissue localization where plasma cells home, and efficient production of complex antibodies with short half-lives or challenging structures for conventional manufacturing (e.g., polyspecific or non-IgG antibodies). Endogenous mAb synthesis would be especially beneficial for chronic disease treatment and, in particular, for tumors showing only partial remission.

Indeed, several strategies have been explored to modify the specificity of immunoglobulins (Igs) produced by B cells, editing the variable (V) region of either the heavy (H) chain only or also of the light (L) chain, and either encoding these chains separately or linking them together.^8^^,^^9^^,^^10^^,^^11^^,^^12^ To achieve the latter goal, we have recently described a strategy in which a single cassette inserted into the IgH locus, with linkers connecting a V_H_ sequence and a complete Ig κ chain sequence, supported the production of a single-chain Full Ig (scFull-Ig) capable of class switching and somatic hypermutation of its V_H_ and V_L_ regions.^9^ This strategy ensured precise and functional single-hit redirection of B cell receptor specificity while preserving its adaptability.

Although promising, such adoptive immunotherapy will require even more stringent safety control systems than for any other drug cell, because B cells are physiologically prone to genetic remodeling. This makes them susceptible to deleterious aberrations eventually leading to uncontrolled proliferation and development of leukemia, lymphoma, or myeloma. Any excessive proliferation of genome-edited therapeutic B cells as well as any overproduction of the therapeutic antibody must therefore be prevented. Ideally, adoptive immunotherapy protocols should include means to switch them off in the case of side effects. Notably for T cells, “suicide gene” systems have been developed, of which the inducible caspase-9-based system (iCasp9) is now widely used.^10^^,^^11^^,^^12^^,^^33^^,^^34^^,^^33^^,^^34^ The iCasp9 gene encodes a chimeric protein binding to the inducer molecule AP1903 (rimiducid).^13^ In the presence of AP1903, the protein dimerizes and initiates an apoptotic cascade.^10^ This system provides an efficient and rapid method of reducing the population of edited cells. The efficacy of iCasp9 compared with other similar approaches has been widely demonstrated, and it has been included in several clinical trials.^11^^,^^14^^,^^15^^,^^16^^,^^17^^,^^18^ However, it is unknown whether this strategy could be applied to therapeutic B cells.

In this study, we describe a strategy to efficiently introduce and implement the iCasp9 gene at the IgH locus together with a single-chain scFull (scFull-Ig) cassette redirected against the HER2 tumor antigen.^9^^,^^19^ Efficient control of the edited cells, either primary B cells or transformed clones from malignant B cell lines, was demonstrated by *in vitro* and *in vivo* experiments. Overall, the results confirm that this approach can ensure an integrated control of the fate of edited B cells and their antibody production.

## Results

### Optimal dosage of AP1903 and evaluation of efficacy in cell lines and primary B cells

**Figure 1:**
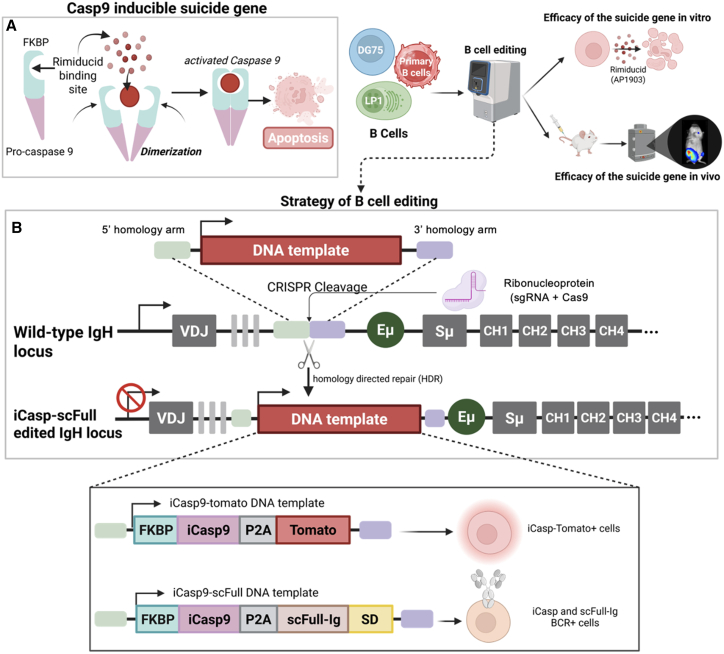
B lymphocyte editing strategy to test the efficacy of the inducible caspase-9 suicide gene (A) Left: schematic diagram of the editing cassette and how iCasp9 is activated by AP1903-induced dimerization. Right: strategy for editing lymphocytes to express iCasp9 and evaluate its induction *in vitro* and *in vivo* (created on Biorender). (B) IgH locus CRISPR-Cas9 editing strategy for insertion of two cassettes encoding iCasp9 in combination with either a tdTomato or an scFull-Ig antibody sequence.

The iCasp9 suicide gene encodes a chimeric protein consisting of pro-caspase-9 and FKBP-F36V binding protein, a mutated variant specifically binding to AP1903. When exposed to AP1903, dimerization of FKBP triggers activation of iCasp9 and thus of a downstream caspase-activation cascade via caspase-3/7, thereby promoting apoptosis (Figure 1A).^13^

To evaluate the iCasp9 system in the B cell lineage, and to easily track cells that have integrated the suicide gene, we first combined it with a tdTomato reporter gene (iCasp9-Tom) which we inserted in the IgH locus downstream of the J_H_ region (Figure 1B). The two proteins were linked by a P2A peptide to be cleaved during translation and support expression of two independent mature proteins (Figure 1B). Using CRISPR-Cas9 technology and previously published IgH locus-specific single-guide RNA (sgRNA) and homology arms, this cassette was inserted into the genome of the IgM^+^ Burkitt’s lymphoma cell line DG75 and the IgG^+^ myeloma cell line LP1. The efficacy of the iCasp9-induced suicide could thus be evaluated in malignant cells corresponding to two different stages of B cell differentiation, mature B lymphocytes for DG75 and plasma cells for LP1. These experiments therefore aimed to assess the efficacy of a suicide gene strategy in the challenging context of aggressive malignant cells and in two scenarios relevant to adoptive B cell-based immunotherapy, where it might be pertinent to edit either the BCR in lymphocytes or only the secreted Ig in plasma cells.

AP1903 is a small synthetic molecule whose safety and pharmacokinetics have been evaluated *in vitro* in animals and in healthy human volunteers with no adverse effects observed in any cell or organ.^10^^,^^15^ To assess specifically on the B cell lineage its lack of intrinsic toxicity, we evaluated *in vitro* its impact on the viability DG75 and LP1 B cell lines edited only with tomato gene without iCasp9. Consistent with data in other lineages, no significant alteration of viability was observed in treated versus untreated cells, using AP1903 at concentrations up to 100 nM (Figure S1).

**Figure 2:**
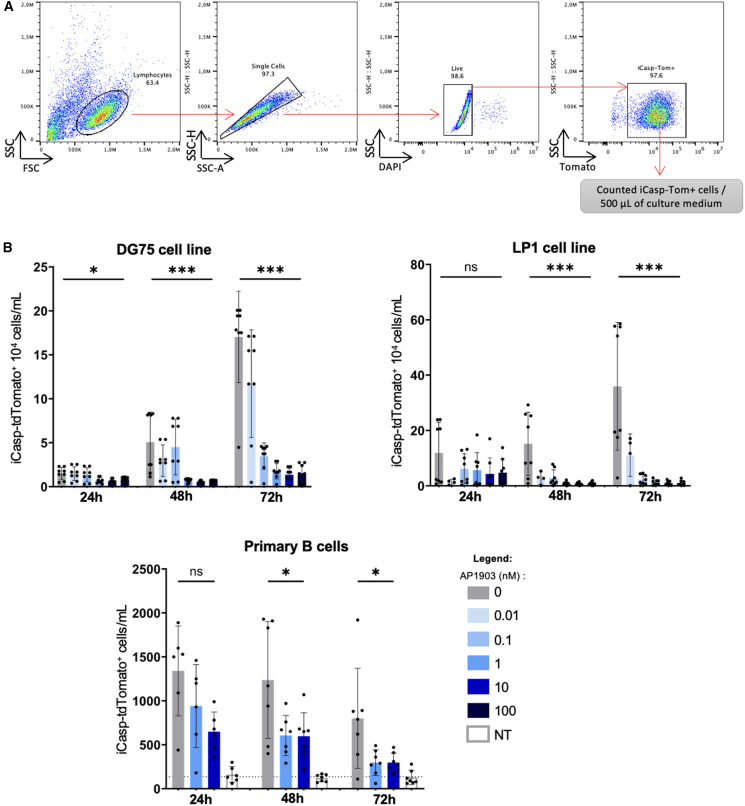
Expression of the iCasp9-tdTomato cassette and efficacy of iCasp9 induction in B lymphocytes (A) Live cells expressing tdTomato were gated as shown (top) and counted after culture of edited DG75 cells (mean ± SD, *n* = 8) (bottom left), and edited LP1 cells (mean ± SD, *n* = 8) (bottom right), with various doses of AP1903 for 24, 48, and 72 h. (B) Live cells expressing tdTomato were gated as above and counted after culture of edited primary B cells with various doses of AP1903 for 24, 48, and 72 h (mean ± SD, *n* = 7). Significant results are indicated: ∗∗∗*p* < 0.0002, ∗*p* < 0.0332, with Mann-Whitney test.

To confirm the efficiency of the iCasp9 system in edited B cells, DG75 and LP1 knock-in (KI) cells homogenously expressing iCasp9 were then sorted by FACS according to their co-expression of the tdTomato reporter gene. Edited cells were then treated with concentrations of AP1903 ranging from 0.01 to 100 nM and collected for analysis of growth and viability of tdTomato^+^ cells by flow cytometry after 24, 48, and 72 h (Figure 2A). A significant and dose-dependent effect was observed for both cell lines, with a progressive decrease in the number of surviving tdTomato^+^ cells, proportional to the increase in AP1903 concentration. The maximum effect was reached after 72 h of treatment in both cell lines. Treatment with 1 nM AP1903 reduced the growth of tdTomato^+^ edited cells by over 70-fold in the DG75 cell line and more than 50-fold in the LP1 cell line (Figure 2B).

Although human primary B cells survive only transiently *in vitro* in normal conditions, we also assessed whether this *in vitro* survival was further reduced by expression and dimerization of iCasp9.^9^ As in previous editing experiments, CRISPR-Cas9-mediated KI was less efficient in primary cells than in cell lines. This partial efficacy hereby provided an internal control and allowed us to compare in the same culture the survival of cells with or without successful KI of the iCasp9-tdTomato cassette (Figure 2B). This showed a significant reduction in the number of Tomato^+^ cells after 72 h of treatment with AP1903, down to a level that was close to the background autofluorescence observed in un-edited non-transfected primary B cells (dotted line in Figure 2B).

### Single-hit genome editing to integrate the iCasp9 gene while redirecting antigen specificity

In a previous work, we developed a genome editing strategy targeting the IgH locus in B cells to replace the V_H_DJ_H_ segment with a KI cassette combining a V_H_DJ_H_ and a complete Igκ chain sequence to produce a scFull-Ig of desired specificity.^9^ The linkers included in the scFull-Ig architecture covalently connect the H and L chain domains to prevent any association of hybrid Igs containing an endogenous L chain.^9^ The iCasp9 gene was thus inserted upstream of this scFull-Ig cassette, with an intercalated P2A element to allow co-expression of both proteins from a single transcript (Figure 1B).

While insertion of this dual iCasp-scFull-Ig cassette was checked by PCR (Figure S2A), we validated that the linkage with iCasp9 did not affect the functionality of the edited Ig in edited B cell lines. Cell cytometry after staining with fluorescent HER2 antigen showed that the dual cassette supported BCR editing of DG75 cells (Figure S2B). Since LP1 only produces Ig in the secreted form, we assessed by ELISA that the culture supernatants from edited LP1 clones did secrete the anti-HER2 scFull-Ig molecule (Figure S2C). These experiments confirmed that the co-expression of iCasp9 and scFull-Ig and the cleavage of the P2A linker neither compromised the Ag specificity of the scFull-Ig nor its functional expression as a BCR or a secreted Ig.

To also assess the functionality of iCasp9 in this architecture, HER2-binding DG75 edited cells were FACS sorted and cloned, while edited LP1 clones secreting anti-HER2 scFull-Ig were also selected with mono- or bi-allelic IgH edition by PCR (Figure S2C).

**Figure 3:**
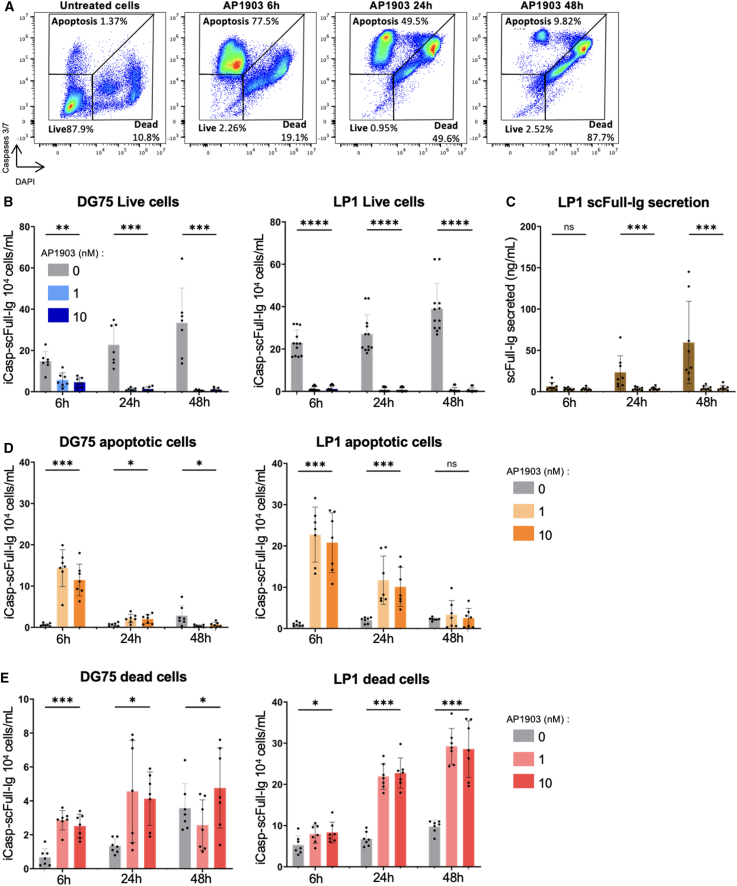
Single-hit editing to insert and evaluate the iCasp9 and scFull-Ig cassettes (A) Cell cytometry strategy for gating live, apoptotic, and dead cells by monitoring caspase-3/7 activation and staining with DAPI at successive stages of AP1903 treatment. (B) Live cells in edited clones of the DG75 cell line (*n* = 7) (left) or the LP1 cell line (*n* = 12) (right) cultured with 0.1 nM AP1903 for 6, 24, and 48 h. (C) Secretion of scFull-Ig followed by ELISA as a marker of the live cell mass, evaluated in the supernatant of edited LP1 clones cultured with 0.1 nM AP1903 for 6, 24, and 48 h (*n* = 8). (D) Early apoptotic cells in edited clones of the DG75 cell line (left) or the LP1 cell line (middle) cultured with 0.1 nM AP1903 for 6, 24, and 48 h. (E) Dead cells in edited clones of the DG75 cell line (left) or the LP1 cell line (middle) cultured with 0.1 nM AP1903 for 6, 24, and 48 h. Mean ± SD. Significant results are indicated: ∗∗∗∗*p* < 0.0001, ∗∗∗*p* < 0.0002, ∗*p* < 0.05, with Mann-Whitney test.

The response to AP1903-induced iCasp9 dimerization was then evaluated *in vitro* in these clones using a cell cytometry fluorescent assay that monitors the activation of caspase- 3 and -7 downstream of caspase-9 (gating strategy shown in Figure 3A). Microscopic and cytometric analysis showed that, compared with untreated cells that rapidly grew, AP1903-treated cells displayed an immediate growth defect (Figure 3B). Consequently, the accumulation of secreted scFull-Ig in the culture supernatants of LP1 clones was decreased by 6-fold after 24 h and 13-fold after 48 h (Figure 3C). In addition to the growth defect, cell cytometry showed that caspase-3/7 activation and apoptosis occurred rapidly, 5 to 7 h after treatment initiation (Figure 3D; Figure S3). Further monitoring of apoptosis and cell death until 48-h post-treatment with 1 nM AP1903 showed that apoptosis culminated after 6 h in both cell lines (with a 22-fold increase), confirming effective iCasp9 activation (Figure 3D), while dead cells started to massively accumulate at 24 h (Figures 3D and 3E). After 48 h of iCasp9 activation by AP1903, the number of remaining viable cells was below 10% for the DG75 cell line, and below 2% for the LP1 cell line. (Figure 3).

### Effect of bi-allelic editing on suicide gene efficacy and resistance

**Figure 4:**
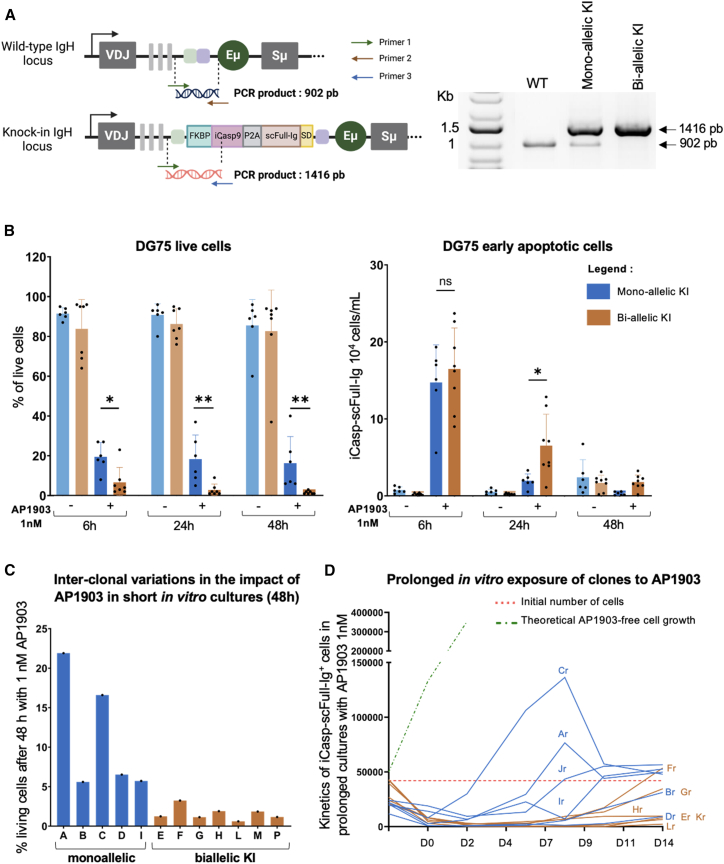
Impact of AP1903 on clones with mono- versus bi-allelic iCasp9 insertion, and development of resistance (A) Schematic of the multiplex PCR assay identifying mono- or bi-allelic IgH insertion of the iCasp9-Ig (left) and electrophoretic gel of showing aspects that correspond to WT DG75 (0.9 kb band), mono-allelic edition (0.9 kb band + 1.5 kb KI band), and bi-allelic edition (single 1.5 kb KI band). (B) Percentage of live cells in the groups of mono- versus bi-allelic clones treated 48 h with AP1903 0.1 nM (left), and quantification of apoptosis (right). Mean ± SD, *n* = 6 (with 6 different clones for each group). Significant results are indicated: ∗∗ *p* < 0.003, ∗*p* < 0.04, with Mann-Whitney test. (C) Quantification of live cells in individual clones after 48 h exposure to 0.1 nM AP1903 for each clone. (D) Quantification of live cells in individual clones and development of resistance after prolonged (up to 14 days) exposure to 1 nM AP1903.

As we also observed different response to iCasp9 induction in different clones of DG75, we investigated whether this was due to copy-number variation, as recently reported in human induced pluripotent stem cells.^20^ In the precise KI strategy used here, copy-number variation could simply correspond to mono-allelic versus bi-allelic editing. Therefore, we compared two groups of edited DG75 clones that either carried the expected KI on a single IgH allele (yielding a KI-specific 1,416 bp PCR fragment) or bi-allelically, i.e., with loss of the 902 bp wild-type (WT) band (Figure 4A). Although individual clones from each group showed variable susceptibility to iCasp9 induction, the susceptibility of bi-allelic clones proved on average higher than for mono-allelic clones, with significantly less-viable and more apoptotic cells after 24 h as well as 48 h with AP1903 1 nM (Figures 4B and 4C).

To assess the long-term fate of cells resisting the iCasp9 activation, cultures of edited cells were prolonged for 2 weeks at a 1 nM concentration of AP1903, renewing the medium every 48 h with freshly prepared AP1903 in order to avoid its degradation. While iCasp9 induction strongly affected cell growth during the first 4 days in all clones (Figure 4D; Figure S4), resistance later developed and some live cells that regrew were finally detectable at day 14 for all clones. Mono-allelic clones started to regrow from day 7 (with a 3.9-fold reduction in doubling time compared with untreated cells), while bi-allelic clones started to regrow from day 9 (with 6.5-fold reduction in doubling time) (Figure 4D). These data therefore demonstrate the significant impact of the iCasp9/AP1903 suicide strategy on overtly malignant B cells, triggering apoptosis and inhibiting growth. However, they also show that a population of AP1903-resistant (AP1903r) cells constantly persists or develops, regardless of whether the gene dosage corresponds to a single or a bi-allelic KI.

### Adaptation of malignant B cells and development of AP1903r cells

To analyze the pathways that can confer iCasp9/AP1903 resistance to edited DG75 cells, we explored the transcriptomic reprogramming selected *in vitro* in eight independent clones cultured either without or with AP1903, until resistant cells regrew (i.e., for over 14 days).

**Figure 5:**
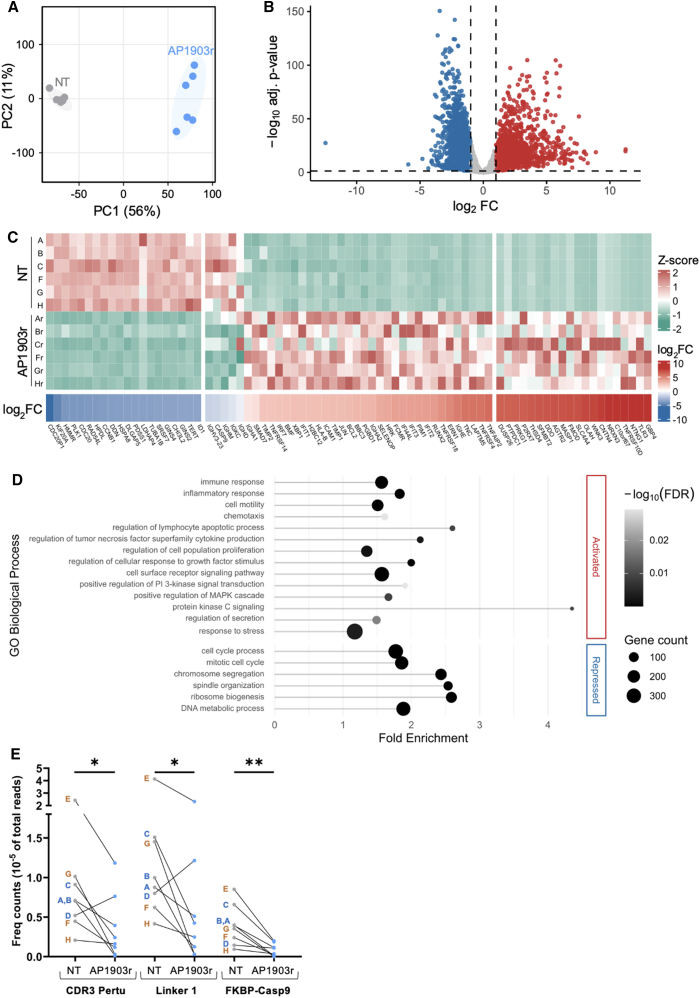
Transcriptomic rewiring in AP1903r clones (A) Principal-component analysis based on normalized counts of all genes. A1903r samples are shown in blue and untreated (NT) samples in gray. (B) Volcano plot showing differential gene expression in AP1903r clones compared with their NT counterparts. Genes with |log_2_FC| > 1 and adjusted *p* < 0.05 are considered differentially expressed: upregulated genes (log_2_FC > 1) are shown in red, downregulated genes (log_2_FC < −1) in blue. (C) Top: heatmap of *Z* scores (gene-wise normalized counts) for differentially expressed genes. Bottom: corresponding logFC for A1903r relative to NT, with increasing red indicating upregulation and increasing blue indicating downregulation. The left panel shows the top 20 most repressed genes, the right panel the top 20 most upregulated genes, and the central panel includes genes of interest related to cell survival, the inserted cassette, or the targeted locus. (D) Selected enriched Gene Ontology (GO) biological process terms among upregulated genes (top panel) and downregulated genes (bottom panel). “Gene count” refers to the number of differentially expressed genes associated with each GO term. “Fold enrichment” indicates overrepresentation compared with the genome background. Significance was assessed using Benjamini-Hochberg FDR correction. (E) Frequency of reads including sequence probes only present in the editing cassette, corresponding to the FKBP-iCasp9 junction, or the pertuzumab IgH chain CDR region, or the linker intercalated between the V_H_ and V_L_ regions, ∗∗*p* ≤ 0.002, ∗*p* ≤ 0.033, with Wilcoxon test.

Six of these eight clones exhibited homogeneous profiles, regardless of whether they had mono-allelic or bi-allelic insertion of iCasp9, and we therefore compared the statistically significant differences in their gene expression (Figure 5). In this homogeneous group, significant changes notably affected the pathways related to cell growth and death, apoptosis, necroptosis, survival, and cell activation signaling pathways (notably PI3K-Akt), and cell cycle and nucleic acid metabolism. In particular in this group, differential expression analyses showed upregulation of several major actors or hubs controlling cell survival, such as TRAILR4 (TNFRSF10D), TRAILR3 (TNFRSF10C), OX40 (TNFRSF4), GITR (TNFRSF18), IRE-1, XBP1, PIM1, BCL2, FCMR, JUN, or SELENOP, which increased from 6- to 240-fold (Log2FC 2.5–7.9).^21^^,^^22^^,^^23^^,^^24^^,^^25^^,^^26^^,^^27^^,^^28^^,^^29^^,^^30^ Some other transcripts highly upregulated in this group were also reminiscent of documented cases of cancer resistance to chemotherapy, including three family members of the interferon-induced proteins with tetratricopeptide repeats (IFIT1, IFIT2, and IFIT3).^31^

Besides this homogeneous group of clones, two less dynamically growing clones (D and E) (Figure 4D) had shifted to different and less homogenous transcriptional profiles (Figure S5).

### Expression of the IgH locus in clones developing *in vitro* resistance to AP1903

The RNA-seq analysis also evaluated whether acquiring resistance to iCasp9/AP1903 affected the expression of the edited IgH locus. Although the relevant genes did not exhibit the highest fold change, they showed significant variation. Notably, the group of six clones with homogeneous transcriptomic profiles demonstrated significantly lower expression of two sequences incorporated into the editing cassette, corresponding to the CASP9 (fold change −3.16) and the IGKC/Cκ gene (fold change −2.0). They also exhibited decreased expression of the expressed V_H_ gene of DG75, IGHV3-23 (fold change −3.23), and of the constant IGHM gene (fold change −2.54).

Conversely, there was a significant increase in the expression of endogenous constant IgH genes located far downstream of the editing site. These included IGHE (fold change 41.7), IGHA2 (fold change 8.54), and IGHA1 (fold change 2.26).

Since this standard RNA-seq analysis sums the expression of KI sequences and homologous endogenous genes, we supplemented it with precise read count enumeration for three probes that were strictly specific to the KI cassette: the linker sequence joining the V_H_ and V_L_ regions of the scFull cassette; its clonotypic V_H_DJ_H_ CDR3 sequence; and the fused sequence at the FKBP/Casp9 junction. These probes are absent from the WT DG75 genome and demonstrated significantly lower expression of the editing cassette in AP1903r clones (Figure 5E) (with expression of FKBP-iCasp9 lower in all clones, and expression of the KI scFull-Ig sequence strongly reduced in seven of the eight clones, but slightly increased in clone D).

### *In vivo* validation of the iCasp-scFull-Ig cassette activity

**Figure 6:**
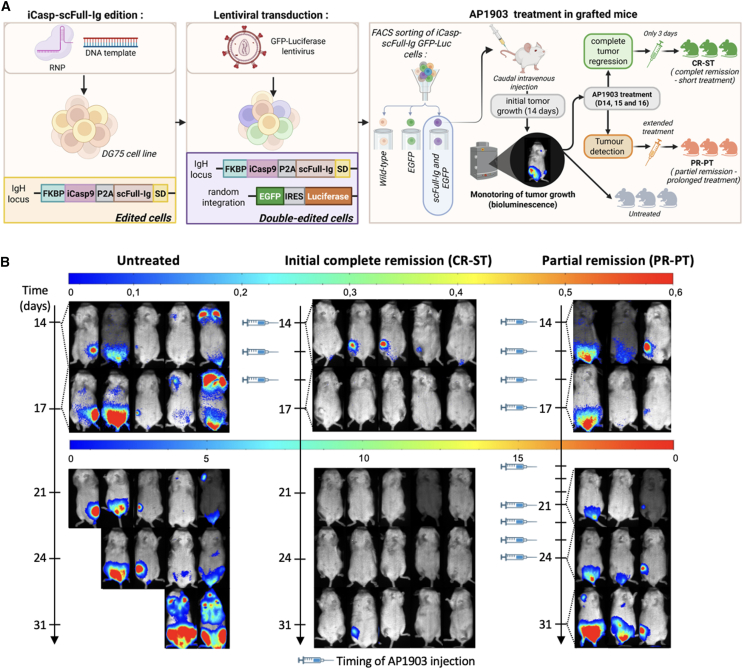
*In vivo* efficacy of iCasp9/AP1903 in mice grafted with [iCasp-scFull-Ig GFP-Luc] lymphoma cells (A) Graphical summary of the strategy for preparing iCasp-scFull GFP-Luc DG75 cells, grafting them into mice, and treating with AP1903. Remission was assessed at day 17. CR-ST designates mice that achieved complete remission after short (3-day) treatment; PR-PT denotes mice in which bioluminescence showed only partial remission, which therefore received prolonged treatment for 2 weeks. (B) Bioluminescence images of each control (untreated), CR-ST and PR-ST mouse with time of AP-1903 treatment.

To investigate the ability of the iCasp9/AP1903 strategy to control edited B cells *in vivo*, we generated a bioluminescent version of the edited DG75 cell line described above, expressing iCasp9 and anti-HER2 scFull-Ig. The cells were then additionally transduced with lentiviral particles conferring expression of eGFP and luciferase, to track tumor development in living animals.^32^ The double-edited GFP^+^/scFull-Ig^+^ cells were FACS sorted (Figures 6A and S6A) and grafted into immunodeficient mice. The mice were then followed by *in vivo* bioluminescence imaging to validate a cohort of animals with effective tumor development at day 14 after tumor cell graft, in which further tumor development could be evaluated with or without treatment of mice by AP1903 at 5 mg/kg, i.e., in accordance with previously published *in vivo* studies carried out for exposing other cell lineages to this drug.^1^^,^^33^ Mice were treated as outlined in Figure 6A, with an initial induction phase of three consecutive daily injections at days 14, 15, and 16, after which bioluminescence imaging was performed at day 17. All treated mice showed either a reduction of the tumor size in comparison with untreated mice, or even an apparently complete remission (CR) (Figure 6B), highlighting the efficacy of the iCasp9/AP1903 system to control the growth of edited B cells *in vivo*. For a longer follow-up, the mice were divided into two subgroups according to their response to therapy as assessed by *in vivo* imaging. For those with apparent early CR, a “stop treatment” (ST) option was chosen (“CR-ST” group). For those with partial remission (PR), prolonged treatment (PT) was undertaken for an additional week (with five injections of AP1903 at days 17, 18, 21, 22, 23, and 24) (“PR-PT” group).

**Figure 7:**
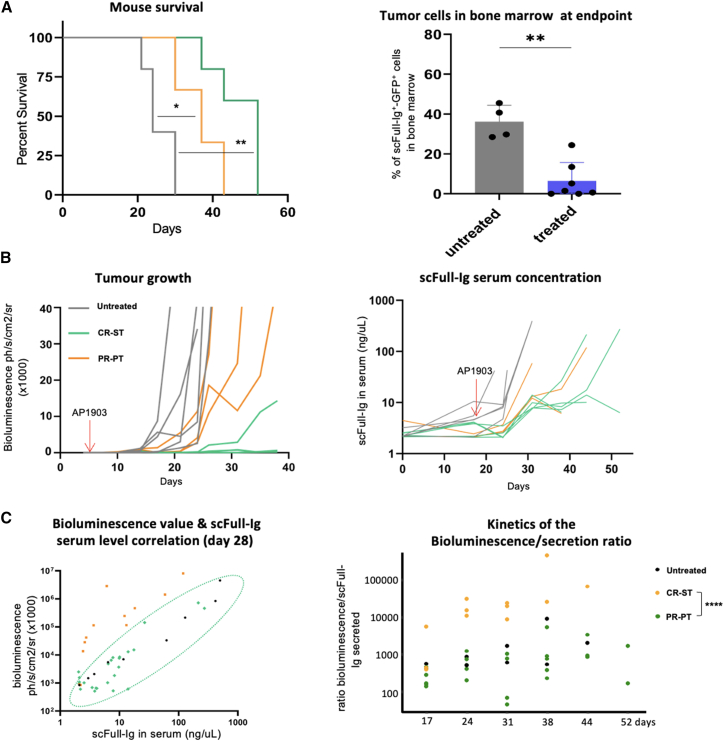
Tumor development and therapeutic Ig secretion in mice with [iCasp-scFull-Ig GFP-Luc] DG75 cells (untreated in black, CR in green, PR in orange) (A) Kaplan-Meier survival curve analysis. ∗∗*p* ≤ 0.002, ∗*p* ≤ 0.033, with Mantel-Cox test (left). Flow cytometry quantification of human malignant B cells in bone marrow from grafted mice at sacrifice. ∗∗*p* < 0.0021, with Mann-Whitney test (right). (B) Kinetics of *in vivo* tumor growth using bioluminescence values (in ph/cm^2^/s/sr) determined for each mouse (left). Kinetics of the serum level of scFull-Ig, as a marker of tumor mass determined by specific ELISA for each mouse (right). (C) Correlation between bioluminescence and scFull-Ig secretion at day 28 (left). Kinetics of the bioluminescence versus scFull-Ig level ratio (right). ∗∗∗∗*p* = 3 × 10^−^^5^ with Wilcoxon test comparing mouse groups and including values of all time points (except day 52 due to insufficient number of mice).

In terms of survival, untreated mice died between days 20 and 30 after the graft of tumor cells. By contrast, the CR-ST group with an early controlled tumor burden following initial treatment, survived for between 38 and 52 days, and the PR-PT group with a partially controlled tumor burden survived for between 30 and 43 days despite prolonged treatment (Figure 7A). Both treated groups exhibited fewer human hCD45^+^/CD19^+^ malignant cells in the bone marrow at the endpoint than untreated animals (Figures 7A and S6B). Despite slowed and/or delayed tumor growth in both treated groups (Figures 7A and 7B), all of the mice finally relapsed when treatment was terminated, and they had to be sacrificed after this due to reaching the endpoints of the animal testing procedure. Bone marrow samples collected from sacrificed mice showed that most tumor cells expressed both GFP and scFull-Ig protein in the untreated as well as in the CR-ST group. By contrast, a heterogeneous and markedly reduced staining by the fluorescent HER2 antigen was noticed by cell cytometry in the PR-PT group. This suggests that prolonged AP1903 treatment had selected cells with downregulated expression of the iCasp9/scFull-Ig cassette, which might participate in the development of AP1903-resistant cells due to decreased abundance of the inducible caspase (Figure S6B).

As the DG75 Burkitt’s lymphoma cell line not only expresses a membrane-anchored BCR but also secretes soluble Ig, we evaluated the serum levels of the scFull-Ig as a biomarker of the tumor mass, similar to the serum monoclonal Ig components paralleling tumor growth in myeloma patients (Figure 7B). Indeed, we observed a direct correlation between this serum level and the bioluminescence score obtained by *in vivo* imaging of tumors (Figure 7C). While this correlation appeared to be similar in the untreated group compared with the CR-ST group with early remission, we also noticed that the PR-PT group that had received a 2-week treatment mapped differently on the linear regression graph at day 28. On average in this group, bioluminescence values were associated to lower levels of scFull-Ig secretion (Figure 7C). Although those two parameters are expected to follow tumor mass expansion, they remained uncoupled over time in the PR-PT group, with an increased ratio of bioluminescence/scFull-Ig secretion at all time points (Figure 7C).

These data show that, *in vivo* as well as *in vitro*, AP1903 strongly affects the growth of B cells expressing iCasp9 but that malignant B cells can develop resistance to this process and that downregulation of iCasp9 expression likely contributes to such acquired resistance. Of note, the expression of a transgene directly linked to the iCasp9 sequence can then undergo a parallel downregulation, which may be of interest in the context of immunotherapy for tuning the expression of associated transgenes encoding therapeutic molecules.

## Discussion

Although promising, the incorporation of genome editing into cell therapy protocols requires safety management to prevent unwanted side effects, and this will be a particular concern once dealing with B cells that are highly exposed to oncogenic transformation. To overcome this issue in the context of future B cell editing therapeutic protocols, we evaluated a CRISPR strategy that could redirect antigen specificity in B cells while providing a control system for the edited cells. In this work, we demonstrate that the iCasp9/AP1903 can be precisely inserted into the IgH locus and appropriately redirected to the B cell lineage, with no leakage affecting cell viability in the absence of the inducer molecule and with efficient and rapid cell death upon AP1903 treatment. We also demonstrate the feasibility of combining the iCasp9 suicide gene and an scFull-Ig cassette that reformats Ag specificity in a single DNA template and a single CRISPR-Cas9 edit, providing a complete system for an immunotherapeutic approach.

Building on a previously described editing strategy that re-target B cells against the HER2 tumor antigen, we incorporated the iCasp9 cassette upstream of the IgH editing template. When expressed in B cell lines and primary B cells, this tandem cassette did not affect the HER2 specificity of the adoptive BCR or its ability to be also expressed as a soluble secreted antibody molecule.

Expression of iCasp9 in this configuration in lymphoma or myeloma cell lines and in primary B cells specifically rendered them sensitive to suicide induction when exposed *in vitro* to AP1903, which rapidly triggered apoptosis and strongly affected the survival and growth of treated cells. While the edited cells consistently showed an immediate response to AP1903, the sensitivity to this drug varied between subclones, revealing a gene dosage effect that made the system even more effective when the integration of the iCasp9 gene was bi-allelic.

Prolonged *in vitro* exposure to AP1903, however, revealed that the control exerted on edited malignant B cell lines and subclones remained incomplete and that resistance to the drug finally emerged in some treated cells, whether they carried a mono-allelic or a bi-allelic KI. This is reminiscent to acquired AP1903 resistance previously documented in lineages and malignant cells of non-B cell origin such as T cells, induced pluripotent stem cells and teratomas, for which resistance to AP1903 could involve downregulation of the iCasp9 transgene expression following methylation of its promoter region?^20^^,^^34^^,^^35^ In such situations with downregulated expression of iCasp9, DNA methylation could thus be partially reversed using 5-azacytidine.^20^^,^^34^^,^^35^

In addition to the *in vitro* assays and to mimic *in vivo* the situation of excessive cell growth that could occur as a side effect of B cell-based adoptive immunotherapy, the DG75 lymphoma B cell line, edited to express an iCasp9/scFull-Ig cassette, was grafted to recipient mice. This experiment demonstrated the *in vivo* efficacy of the iCasp9/AP1903 strategy against cells representative of Burkitt’s lymphoma, one of the fastest growing human cancers, with a cell doubling time of 1–2 days and rapid spread to the liver, spleen, and bone marrow.^36^ Despite this aggressiveness, curative treatment with AP1903 was possible in mice with previously established tumors, for which complete or PR occurred and survival was improved. Reduced tumor burden was consistently assessed by *in vivo* imaging of tumors, serum levels of the edited monoclonal Ig and finally by reduced bone marrow tumor cell infiltration seen at sacrifice. However, similar to the *in vitro* assays and likely due to reduced expression of the iCasp9 KI cassette, all mice only treated with AP1903 relapsed.

Our *in vitro* study allowed us to analyze in detail how malignant B cell clones had acquired resistance to AP1903. Similar pathways involved in a main group of six homogeneous clones. These mostly included the upregulation of genes involved in various cell death and survival pathways. TRAILR3, TRAILR4, and BCL2 are known to be anti-apoptotic factors.^21^^,^^27^ IRE1 and XBP1 are master regulators of the unfolded protein response and play a role in promoting the survival, growth, and differentiation of immune cells exposed to ER stress.^25^^,^^37^ TNFRS14/OX40 is known for its role in T cell expansion, while TNFRSF18 (GITR) regulates apoptosis in immune cells, notably protecting T cells from TCR-induced apoptosis.^38^ PIM1 is a kinase and well-known inducer of cell growth and survival.^26^^,^^39^ The FCMR/TOSO membrane receptor promotes survival and inhibits apoptosis in malignant B cells, and SELENOP (selenoprotein P) is an inhibitor of ferroptosis.^30^ Interestingly, several of these pathways are accessible to clinically available small molecules inhibitors such as venetoclax for the BCL2 pathway and PIM-kinase inhibitors.

Of interest, some genes broadly known for a role in resistance to chemotherapy are also upregulated in this main group of AP1903-resistant clones. Notably, HIST1H2BK was reported to inhibit 5-FU-induced apoptosis by upregulating A2M transcription and activating the PI3K/Akt pathway, thereby promoting cell survival.^40^ Tumor progression and resistance to chemotherapy have also been correlated with increased expression of the IFIT1-3 molecules.^31^

Besides this main pathway, it is also notable that two individual clones followed other strategies of transcriptomic rewiring.

Collectively, the iCasp9/AP1903 strategy can thus potently control edited B cells. By contrast, it is unlikely to eradicate by itself the potential malignant complications of adoptive B cell therapies. However, since other B cell-ablating options are available, the iCasp9/AP1903 strategy appears to be a potent neoadjuvant therapy and a valuable safety switch to combine with B cell edition.

Beside malignant transformation, other less dramatic side effects of adoptive immunotherapy have been reported with T cells. These can result directly from the function of these cells, with excessive cytokine release, inflammation, or autoimmune complications. The iCasp9/AP1903 strategy has been reported to be valuable for modulating such side effects.^33^^,^^41^ Similarly, edited B cells used to deliver a therapeutic molecule will require a safety rheostat enabling this delivery to be lowered or switched off, and the iCasp9/AP1903 strategy could then answer this need. Interestingly, in our study of tumor-bearing mice with PT, the level of the secreted Ig got shifted downwards by comparison to the bioluminescence score, strongly suggesting a downregulated expression of the single gene cassette where both the iCasp9 and the scFull-Ig sequences are combined. The iCasp9/AP1903 strategy may therefore not only help to control the number of edited B cells growing *in vivo*, but also provide a means to regulate their antibody production on a per-cell basis by selecting cells in which the expression of the editing cassette is down-modulated.

Altogether, this study shows the efficacy in human B cells of the iCasp9/AP1903 system. It appears as an effective neoadjuvant therapy for lymphoma or myeloma malignancies arising from adoptively transferred edited B cells. Albeit attractive, this iCasp9-induced cell death strategy has limitations, which may involve both iCasp9 gene silencing and the development of anti-apoptotic pathways by malignant cells. As such, AP1903 would certainly not be sufficient as a monotherapy but would be of great interest for synergizing with conventional chemotherapy and immunotherapy in order to treat malignant complications of B cell-based therapy. This situation is also reminiscent to that reported in the 293T and HeLa cancer cell lines, where the effect of AP1903 was enhanced by combining it with an additional pro-apoptotic strategy.^42^

Our data are consistent with previous studies showing that the efficacy of suicide induction increases with iCasp9 gene expression.^14^ Although we used a pVH promoter, which is expected to be optimal in the context of a targeted IgH locus, improving the homology-directed repair recombination (HDR) rate to efficiently obtain bi-allelic insertions may further enhance KI cassette expression. However, we observed that double gene dosage only delayed but did not prevent the selection of AP1903r cells.

Aside from the rare occurrence of malignant transformation of therapeutic cells, adverse effects related to their function are possible complications of cell therapy. The ability to control the mass and expansion of transferred cells using a small, specific, non-toxic molecule, such as AP1903, will be invaluable in defining guidelines for adoptive B cell immunotherapy. We additionally observed that those edited cells surviving in the presence of AP1903 can be transcriptionally inhibited at the level of the iCasp9 but also the associated therapeutic sequence. In this context, the iCasp9 gene appears not only as a suicide gene but also a means to control the dosage of the therapeutic molecule by combining both genes in a single KI. Although additional *in vivo* studies are needed to confirm that Ig production by edited primary B cells can be precisely controlled through the iCasp9/rimiducid system, having a tool to regulate both per-cell Ig output and the total number of Ig-secreting cells is highly advantageous. This approach thus shows promise for adoptive immunotherapy, with the option to hereby build therapeutic cells that behave as tunable micropharmacies.

In conclusion, this study demonstrates the adequacy of AP1903/iCasp9 strategy to control the expansion of genome-edited B cells either *in vitro* or *in vivo* and it additionally suggests that the same strategy could enable to modulate the production of an adoptively delivered therapeutic Ig molecule in future B cell-based immunotherapies.

## Materials and methods

### Plasmid construction and donor DNA preparation

Plasmids containing donor DNA sequences were constructed using the NEBuilder HiFi Kit (New England Biolabs). Two plasmids, iCasp9-tdTomato and the HER2-targeted iCasp9-scFull-Ig, were assembled using blocks from previously published plasmids.^11^ Donor DNAs for CRISPR-Cas9 were amplified from these plasmids by PCR using Taq GXL (Takara Bio) and purified using the NucleoSpin Gel and PCR Clean-up Kit (Macherey-Nagel). For each step, DNA fragment sizes were first validated by electrophoresis on a 1% agarose gel and full sequences were determined.

### Cell lines

The DG75 IgM^+^ human Burkitt’s lymphoma cell and the LP1 IgG^+^ myeloma cell line were grown in RPMI GlutaMAX medium (Gibco) supplemented with 10% fetal bovine serum (FBS) (Gibco), 1 mM sodium pyruvate (Gibco), and 1 mM minimal essential medium non-essential amino acids solution (Gibco) at 37°C with 5% CO_2_.

### Primary B cells

Buffy coats from healthy volunteers were collected with their informed consent at the Etablissement Français du Sang (Rennes, France). Circulating primary B cells were positively selected using the StraightFrom Buffy Coat CD19 Microbead Kit (Miltenyi Biotec). B cells were then cultured at 0.75 × 10^6^ cells/mL in RPMI 1640 GlutaMAX medium (Gibco) supplemented with 10% FBS (Gibco), 1 mM sodium pyruvate (Gibco), and 1 mM minimal essential medium non-essential amino acids solution (Gibco). Cells were stimulated for the first 4 days with 1 μg/mL CpG oligodeoxyribonucleotide (CpG 2006; Miltenyi Biotec), 2.4 μg/mL F(abʹ)2 fragment goat anti-human IgA + IgG + IgM (H + L) (Jackson ImmunoResearch), 50 U/mL recombinant IL-2 (R&D Systems), and 100 ng/mL recombinant human soluble CD40L (Immunex). On day 3, 5 ng/mL IL-10 (R&D Systems) was added to the culture medium. On day 4, the cells were washed and transferred (at 0.5 × 10^6^ cells/mL) in a plasmablast differentiation cocktail containing 50 U/mL IL-2 (R&D Systems), 5 ng/mL IL-4 (R&D Systems), and 12 ng/mL IL-10 (R&D Systems).

### Gene editing

For CRISPR-Cas9 transfection, cells were seeded at a concentration of 0.2 × 10^6^ cells/mL the day before transfection to ensure that they were in an exponential growth phase on the day of transfection. CRISPR-Cas9 cleavage was achieved by transfection of a ribonucleoprotein (RNP) complex formed by assembling at 37°C for 15 min 100 pmol of Cas9 (IDT) and 500 pmol of sgRNA (Synthego), which cleaved the following site (in the intronic region following JH6 and preceding the IGHM gene): GGAAAGAGAACTGTCGGAGT; human genome GRCh38, CHr14, 105862762–105862781).

The DG75 cell line was transfected with the Amaxa Nucleofector 1 device (Lonza) using the Cell Line Nucleofector V kit (Lonza). A total of 3 × 10^6^ cells was centrifuged at 500 × *g* for 5 min and then resuspended in 100 μL electroporation buffer. RNP and donor DNA (2 μg) added to the cell suspension were transfected using the “X-01” program. The cells were then cultured in 5 mL of complete medium in six-well plates.

Transfections of LP1 and primary B cells were performed using the ATx device (MaxCyte). A total of 3 × 10^6^ cells was centrifuged at 90 × *g* for 10 min, washed with 5 mL electroporation buffer (MaxCyte), and resuspended in 25 μL electroporation buffer. RNP (250 pmol sgRNA and 50 pmol Cas9) and donor DNA were added to cells for transfection using the “THP1” program for LP1 and “B cell 2” for primary B cells. Processing assemblies were placed at 37°C for 30 min with the addition of 0.5 U pulmozyme (Roche, 2,500 U/2.5 mL). LP1 cells were then cultured in 5 mL complete medium. Primary B cells were resuspended in 2 mL medium with cytokines in 24-well plates with Alt-R HDR Enhancer V2 (IDT). After 24 h, the HDR enhancer was removed and the washed cells were further cultured for 24 h at 32°C.

### Flow cytometry

Flow cytometry evaluations were done on samples of 0.3 × 10^6^ cells washed with PBS. Staining with DAPI (Invitrogen) was used in order to exclude dead cells. HER2 binding was evaluated on cells after washing with PBS + 3% FBS and labeling for 30 min on ice with a pre-assembled fluorescent complex of 20 ng human HER2-biotin (R&D Systems) and 10 ng streptavidin-APC (eBioscience) or PE-CF594 (BD Horizon). Cells were analyzed using a CytoFLEX cytometer (Beckman Coulter) and the FlowJo software.

### Sorting, subcloning, and PCR validation of edited cells

Homogeneous populations of edited cells were sorted using the BD FACS Sorter (BD Biosciences), and then further either cultured in bulk format and maintained at a concentration of 0.5 × 10^6^/mL, or distributed as individual cells in culture microplates in order to grow subclones deriving from a single edited cell.

### Validation of the DNA template insertion

DNA was extracted from 0.3 × 10^6^ edited cells using the QuickExtract DNA Extraction Solution (Biosearch Technologies), which included proteinase K treatment for 6 min at 65°C and proteinase K inactivation for 5 min at 98°C.

DNA samples were analyzed using Purple Taq (Ozyme) in a multiplex PCR assay based on three primers, both of them flanking the insertion site and one located in the inserted cassette. This assay amplified in parallel specific bands corresponding to the edited and the WT IgH locus (with respective sizes of 1,416 and 902 bp). For cloned cells, this assay thus also distinguished the WT configuration from a mono- or bi-allelic edition. The primers used are listed in Table S1.

### ELISA

ELISA assays were used to detect the secreted form of the scFull-Ig molecule. MaxiSorp Clear Flat-Bottom 96-well plates (Nunc) were coated with 10 μg/mL streptavidin (Sigma) overnight at 4°C and blocked with PBS containing 3% BSA (Sigma) for 1 h at 37°C. Next, 0.5 μg/mL biotinylated human HER2/ErbB2 (Sino Biological) was added and the mixture was incubated at 37°C for 1 h. After washing plates, samples were incubated for 2 h at 37°C, followed by washings and a secondary incubation with an alkaline phosphatase-coupled goat anti-kappa human antibody (Southern Biotech) for 1 h at 37°C. Bound antibodies were revealed with alkaline phosphatase substrate p-nitrophenyl phosphate (Sigma) for 15 min, before stopping the reaction with 3 N NaOH. Absorbance was read at 405 nm, and concentrations were calculated by interpolation using GraphPad software (Prism).

### RNA-seq preparation and analysis

RNA from DG75 iCasp-scFull-Ig clones and their AP1903r derivatives were extracted with TriZOL and their quality was controlled by electrophoresis on a TapeStation (Agilent) before sending to BMKGene (Biomarker Technologies) for library preparation and sequencing in 2× 150 bp reads using the Illumina NovaSeq X platform.

Sequencing data were processed with the nf-core/rnaseq pipeline (v.3.18.0). Within this pipeline, quality control was performed using FastQC (v.0.12.1); adapter trimming was carried out with Trim Galore (v.0.6.10) and Cutadapt (v.4.9); read alignment to the GRCh38.p14 reference genome was done using STAR (v.2.7.11b); and transcript quantification was performed using Salmon (v.1.10.3) with the Gencode basic release 48 annotation (Ensembl 114). Genes with an average expression of fewer than one read per sample were prefiltered prior to differential expression analysis. This analysis was conducted using DESeq2 (v.1.42.1), with subsequent filtering of low-expressed genes using HTSFilter (v.1.42.0) and multiple testing correction applied via the false discovery rate (FDR) method.

Functional enrichment analysis was then performed using g:Profiler on both upregulated genes (log2FC > 1 and adjusted *p* < 0.05) and downregulated genes (log2FC < −1 and adjusted *p* < 0.05), based on Gene Ontology biological process terms. Significance was assessed using the FDR correction.

Precise enumeration of reads specific for the KI cassette, expressed as frequencies by reference to the total number of reads in each sample, was done by counting the occurrences of the three following probes:

FKBPCasp9, TGGAGCTTCTAAAACTGGAATCTGGCGGTGGCTCCGGAGTCGACGGATTTGGTGATGTCGGT;

CDR3 Pertu, TGCGCGAGGAACCTGGGTCCTTCCTTCTACTTCGACTACTGG.

Linker 1, GGTGGTGGTGGTTCTGGTGGTGGTGGTTCTGGCGGCGGCGGCTCCAGTGGTGGTGGATCC.

### *In vitro* evaluation of the AP1903 treatment

The small molecule AP1903 (MedChemExpress) was used at concentrations ranging from 0.001 to 100 nM to induce iCasp9 dimerization in edited cells seeded at 0.3 × 10^6^/well in 48-well plates. Wells were then collected at successive time points for cytometric follow-up of cell growth, of the edited antigen specificity (by staining cells with fluorescent HER2 antigen), of cell apoptosis (using the CellEvent caspase-3/7 detection reagent from Invitrogen), and of cell viability (as indicated by DAPI staining).

### Lentiviral transduction of the GFP-luciferase gene

iCasp-scFull DG75 KI cells (1 × 10^6^) were spinofected at 500 × *g* for 30 min at 37°C in medium containing 8 μg/mL protamine sulfate in the presence of a lentivirus containing encoding eGFP and luciferase (GFP-Luc, Addgene, no. 46793). Cells remained in lentiviral precaution for 7 days and were then sorted by FACS as described above.

### Mouse models

All *in vivo* experiments were performed in accordance with animal ethics regulations and all protocols were approved by the French Ministry of Research in accordance with European Union regulations (APAFiS 50804).

### Transplantation of DG75 iCasp-scFull-Ig-GFP-Luc cells

BRGS mice (BALB/c RAG2−/− IL2γC−/− SIRPα.NOD) were transplanted caudally with 0.5 × 10^6^ DG75 KI iCasp-scFull-GFP-Luc in 100 μL PBS. Mouse tails were heated in water to a maximum of 40°C to dilate the vein before slow intravenous injections.

### Bioluminescence imaging

Mice were injected intraperitoneally with D-luciferin (Interchim) at 15 mg/mL. They were then anesthetized with 2% isoflurane in oxygen. Imaging was performed twice weekly to monitor tumor growth using bioimaging (PhotonIMAGER Optima system, Biospace Lab). Image processing and quantification in photons/cm^2^/s per steradian (ph/cm^2^/s/sr) were performed using M3 vision software (Biospace Lab).

### iCasp9 activation by AP1903 treatment

Fourteen days after intravenous injection, mice were imaged by bioluminescence and only those mice with successful engraftment were included in the study. Two groups of untreated versus AP-1903-treated mice were formed to evaluate the iCasp9/AP1903 suicide strategy *in vivo*. A first round of induction therapy included daily intraperitoneal injections of 100 μg AP1903 (in 150 μL) (i.e., 5 mg/kg), solubilized in corn oil (MedChemExpress), for 3 consecutive days. After this short treatment, another imaging session allowed to split the treated mice into two subgroups: treatment was discontinued in highly responsive mice with apparent CR that no longer exhibited any bioluminescence signal, in a second subgroup of mice with PR mice that retained a detectable tumor by bioluminescence imaging, AP1903 injections were prolonged for a total duration of 2 weeks.

### Blood sampling and analysis

Once a week, 100 μL of blood was collected sub-mandibularly from mice grafted with tumor cells. Samples were centrifuged at 600 × *g* for 10 min and plasma was collected. HER2 antibody-specific ELISA were performed on plasma to evaluate the secretion of scFull-Ig.

### Statistical analysis

Data are presented as mean ± SD with 95% confidence intervals. Statistics were done using GraphPad Prism 8 software. Significance is based on Mann-Whitney tests (ns, not significant; ∗*p* < 0.00332, ∗∗*p* < 0.0021, ∗∗∗*p* < 0.0002, ∗∗∗∗*p* < 0.0001), and for survival curve on Mantel-Cox test (ns, not significant; ∗*p* < 0.033, ∗∗*p* < 0.002, ∗∗∗*p* < 0.001).

## Data and code availability

The data that support the findings of this study are available on request from the corresponding author.

## Acknowledgments

We thank Elise Dessauge and Thomas Lejeune for their help with mice. Cell sorting was performed at the Biosit Flow Cytometry and the CytomeTRI cell sorting facility (Université de Rennes/UMS6480 Biosit). We thank the staff from the ARCHE core facility for animal care (Université de Rennes/UMS6480 Biosit). J.L. was supported by a joint fellowship from 10.13039/501100010481Etablissement Français du Sang and Agence Nationale Recherche Technologie (ANRT). This work was supported by 10.13039/501100001665Agence Nationale de la Recherche (grants PEPR THERA-B and ANR-23-CE17-0024-01) and by Association Leucémie Espoir 22.

## Author contributions

Investigation, J.L., M.C., A.-L.T., N.U., O.D., G.N., Y.D., and M.C.; validation, J.L. and L.D.; methodology, J.L., M.C., A.-L.T., L.D., N.U., O.D., G.N., C.F., Y.D., and M.C.; formal analysis, J.L.; visualization, J.L.; writing, J.L., Y.D., and M.C.; conceptualization, C.F., Y.D., and M.C.; supervision, Y.D. and M.C.

## Declaration of interests

The authors declare no competing interests.

## Supplemental information

Document S1. Figures S1–S6 and Table S1

Document S2. Article plus supplemental information
