# Supplementary material for: Using the iCasp9 suicide strategy to control the growth and function of genome-edited B cells with redirected antigen specificity
Source: Mol Ther Oncol. 2025 Nov 22;33(4):201104. doi: 10.1016/j.omton.2025.201104 (PMC12721158; doi:10.1016/j.omton.2025.201104)
Supplement: Document S1. Figures S1–S6 and Table S1 [file mmc1.pdf]

OMTON, Volume *33*

Supplemental information

Using the iCasp9 suicide strategy to control the growth and function of genome-edited B cells with redirected antigen specificity

Jenny Léonard, Marine Cahen, Anne-Laure Tanguy, Laurent Deleurme, Natsuko Ueda, Ophélie Dézé, Grégory Noël, Maiwenn Pineau, Christophe Ferrand, Yannic Danger, and Michel Cogné

Table S1. Sequences of primers used in this study

	Primer	sequence
Primers flanking the IgH knock-in (Figure 4A)	IgH_enhancer_Rev	TGCTTCCAGCTTCGCTCAAT
	IgH_J6_courst_av_BH_For	GGTCACCGTCTCCTCAGGTA
	iCasp9_IgH_3primer	AGCAAAGCCAGCACCATTTTC
Primers for PCR amplification of DNA template	415_tCTS_368_For	TGGCGGGACTAGTGGCCCTTAGAGAACTGTCGGAGTGG GAAGAATGGCCACTCTAGGGC
	416_tCTS_368_Rev	TGGCGGGACTAGTGGCCCTTAGAGAACTGTCGGAGTGG GAGCTTGCTTTGGCCTCAATT

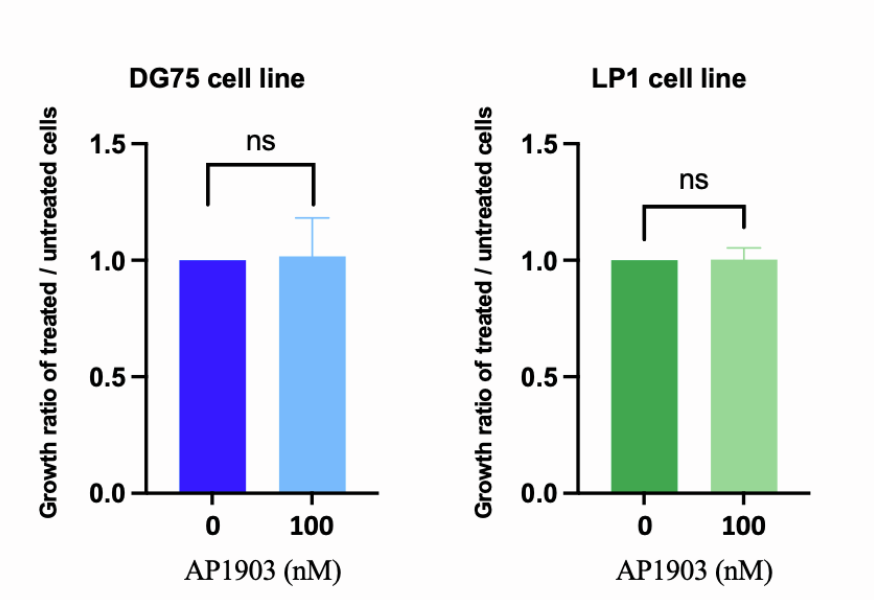

Figure S1: Effect of AP1903 on the growth of un-edited cells from the DG75 and LP1 cell lines. ns, not significant with Mann-Whitney test (n=8).

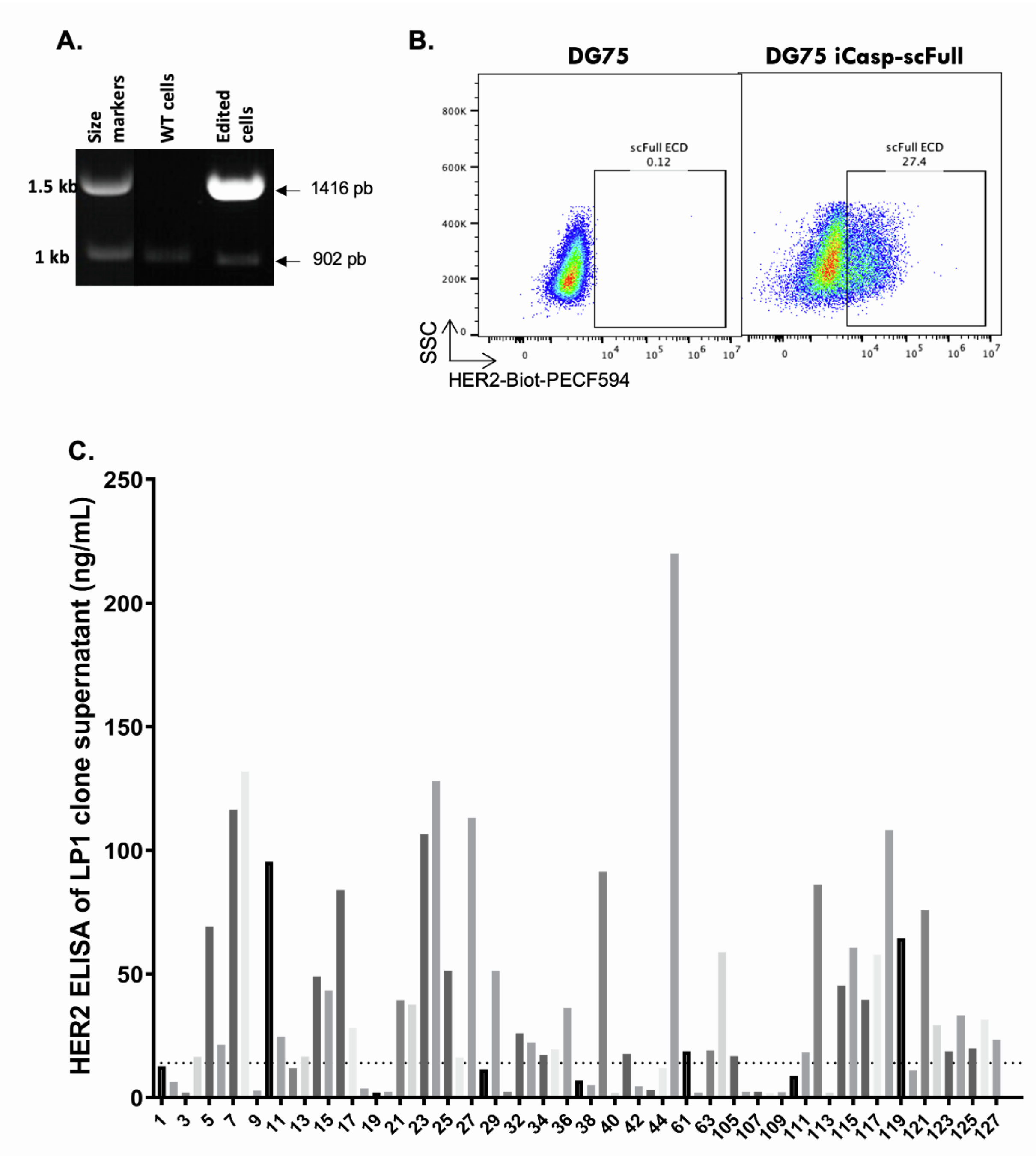

Figure S2: A. PCR bands obtained with the multiplex PCR to detect a 0.9 kb WT or a 1.5 kb edited band after insertion of the iCasp9/scFull-Ig cassette in the IgH locus. **B.** Flow cytometry shows DG75 cells stained with HER2-Biotine-streptavidin-PECF594, after BCR edition and KI of the iCasp9/scFull-Ig cassette. **C**. ELISA evaluation of anti-HER2 scFull-Ig secretion by various clones of edited LP1 cells.

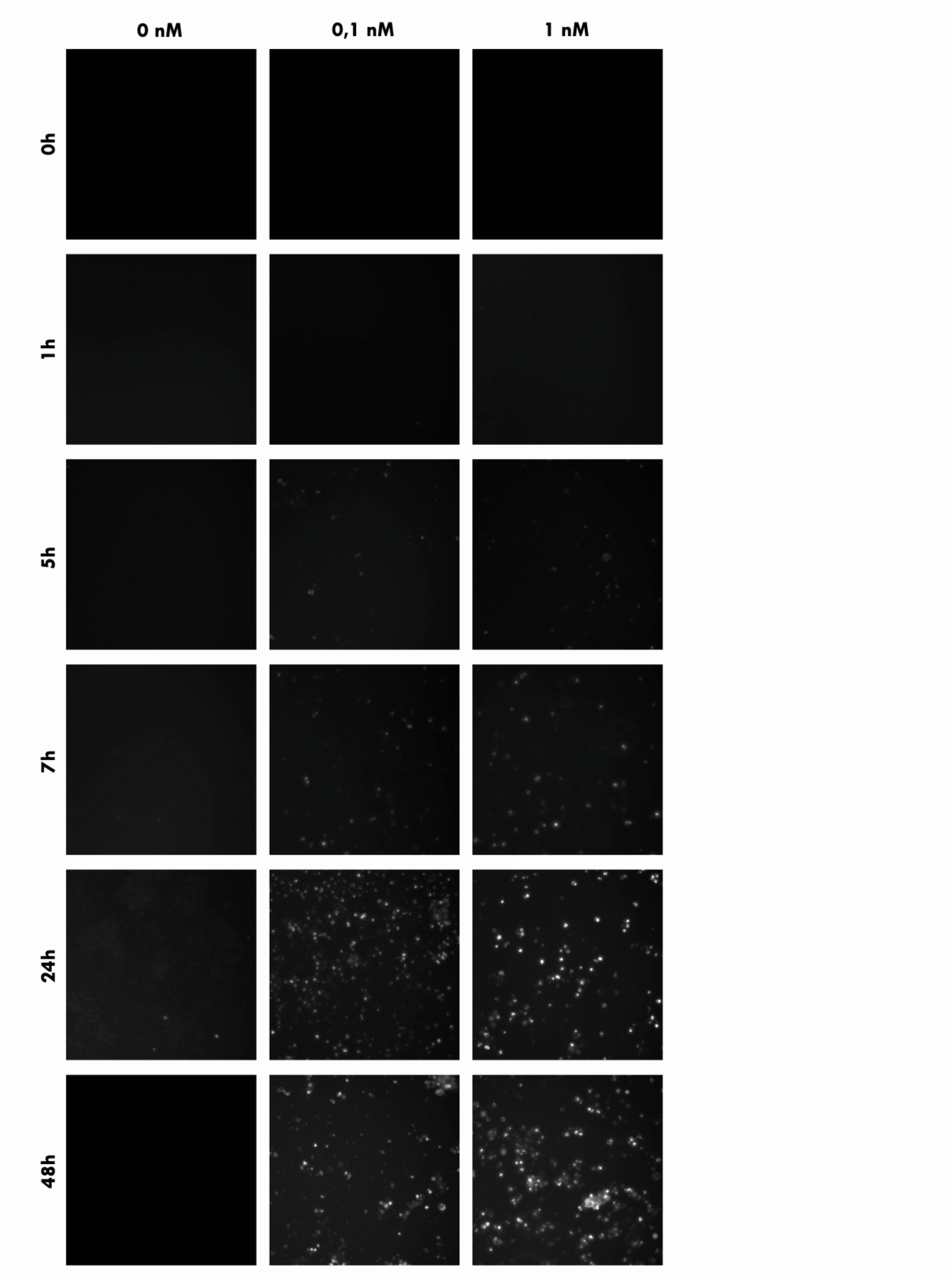

Figure S3. Caspase 3/7 fluorescence after *in vitro* exposure to 0.1 or 1 nM AP1903, followed over time from T0 to 48h in DG75 cells carrying the iCasp9/scFull-Ig cassette inserted into the IgH locus.

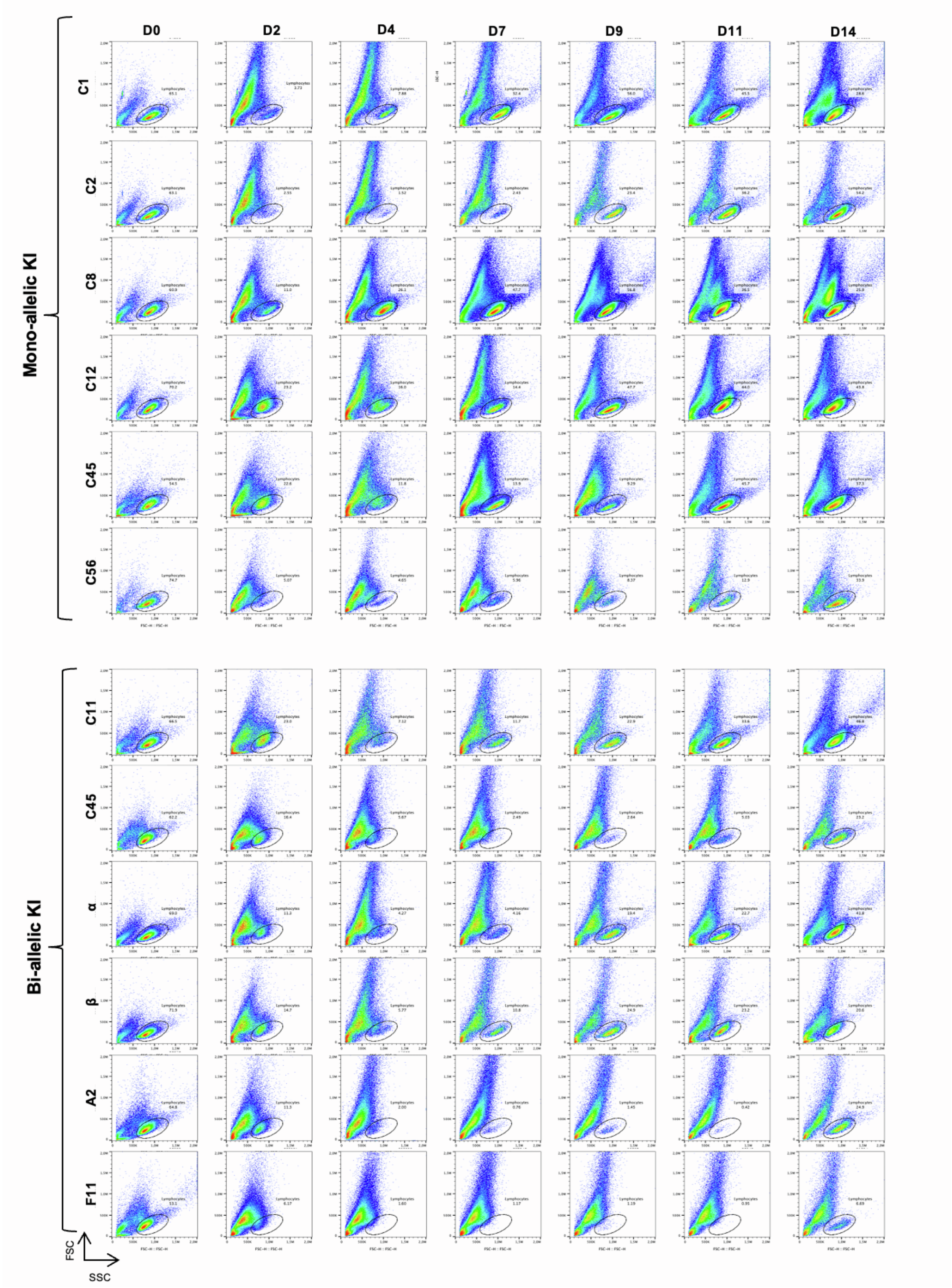

Figure S4. Flow cytometry data from several mono- and bi-allelic KI DG75 clones, after continuous treatment with AP1903 and cell sampling on days: 0, 2, 4, 7, 9, 11, and 14.

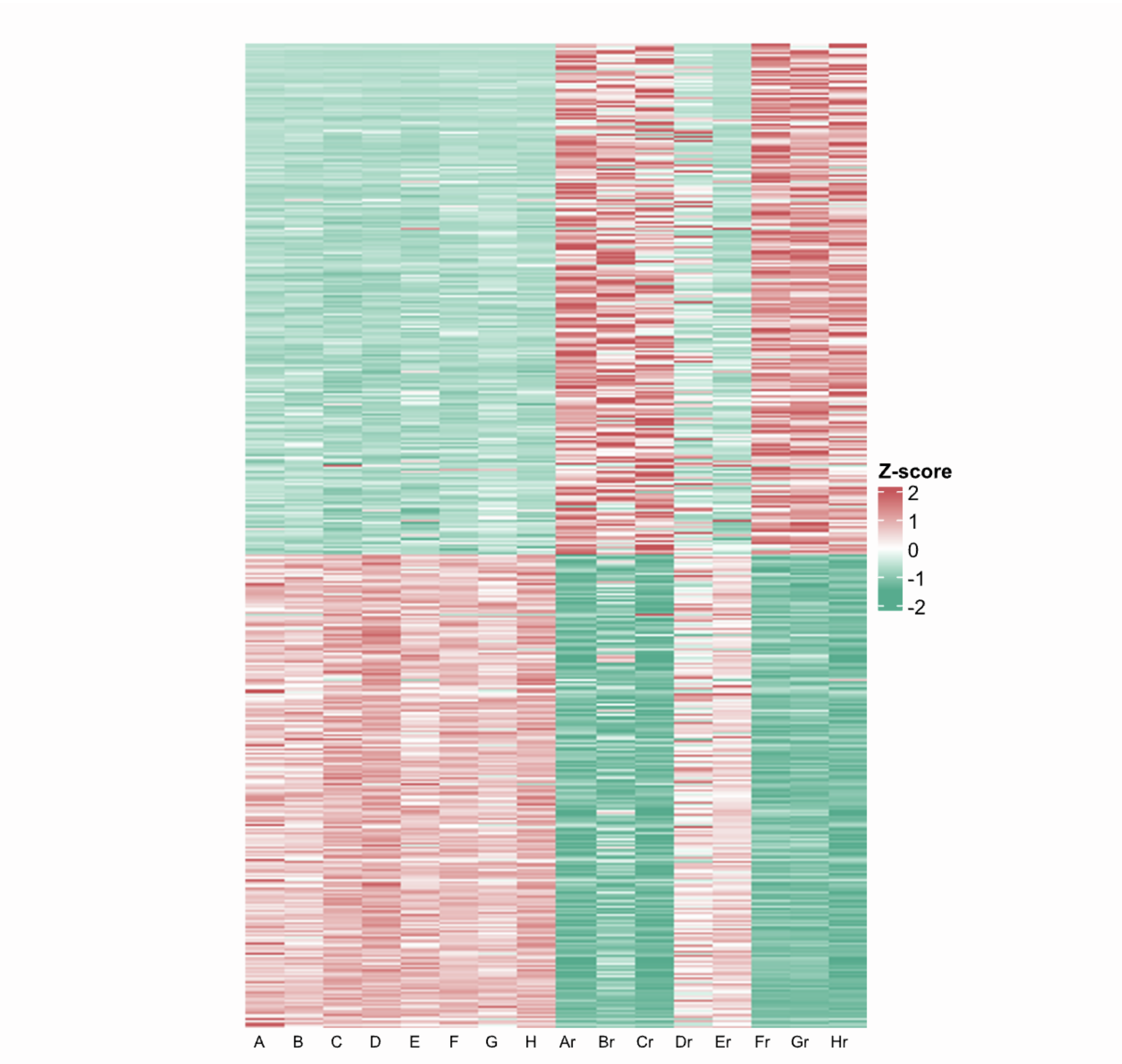

Figure S5. Heatmap showing z-scores (row-wise normalized counts) of all differentially expressed genes (|log2FC| > 1 and adjusted *p*-value < 0,05). Data are shown for all the eight clones A to H, and their AP1903r derivatives (Ar to Hr).

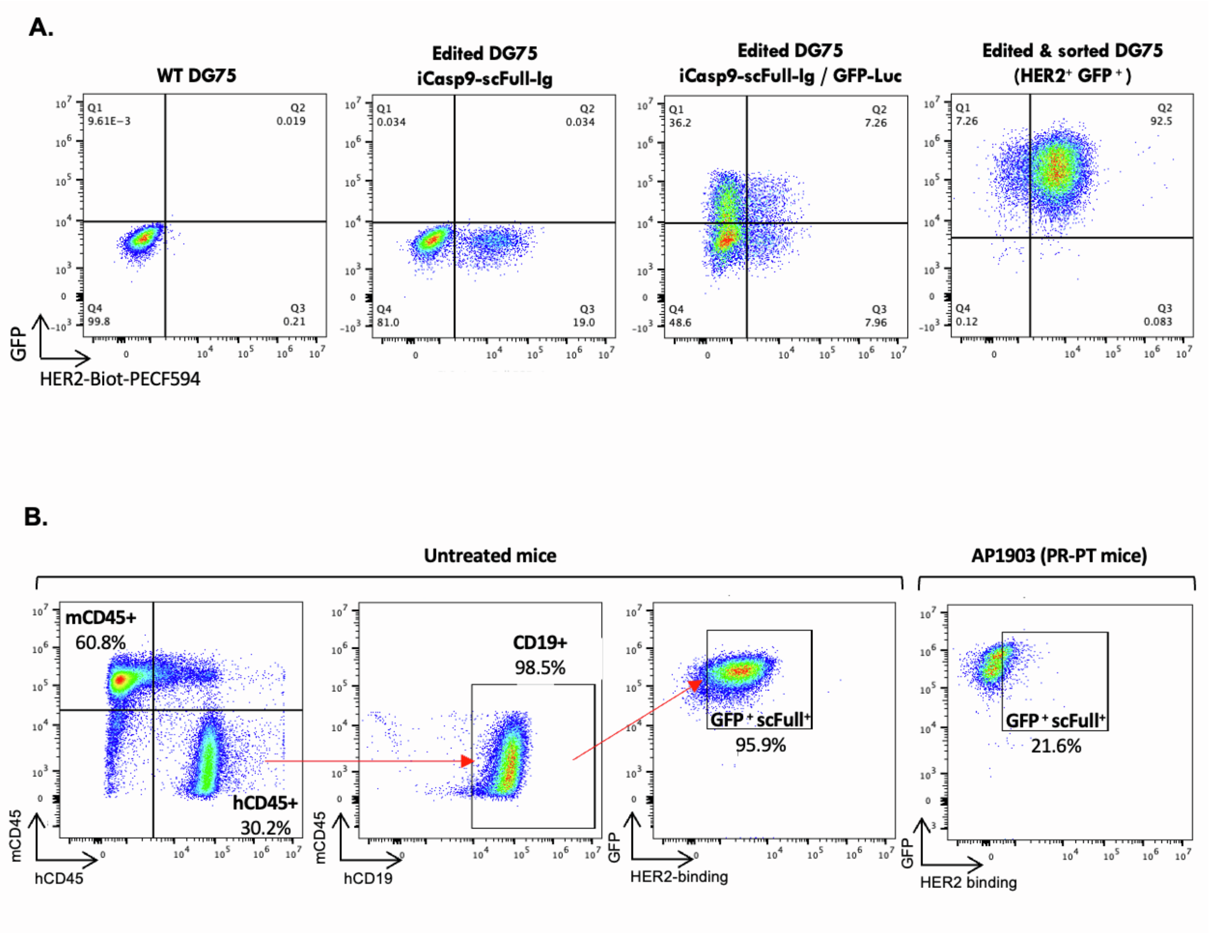

Figure S6. A. Flow cytometry gating strategy for edited DG75 cells with the iCasp9-scFull-Ig KI stained with HER2-Biotine-streptavidin-PECF594, after spinofection with the GFP-encoding lentivirus. **B.** Flow cytometry identification of GFP$^+$ edited (HER2-binding) DG75 cells in bone marrow from mice grafted with tumor cells (*first 3 graphs from the left showing the gating strategy*), and eventually treated with AP1903 (*right*).
